# Evaluating the efficacy of endodontic microsurgery for teeth with an undeveloped root apex and periapical periodontitis after nonsurgical treatment failure

**DOI:** 10.1186/s12903-023-03117-5

**Published:** 2023-06-22

**Authors:** Yumu Tang, Ke Xu, Yumao Chen, Le Lu

**Affiliations:** 1 Department of Endodontics, Suzhou Stomatological Hospital, Suzhou, 215005 China; 2 Department of Orthodontics, Suzhou Stomatological Hospital, Suzhou, 215005 China

**Keywords:** Abnormal central cusp, Periapical periodontitis, Apical foramen, Endodontic microsurgery, Immature teeth

## Abstract

**Aim:**

To determine the efficacy of endodontic microsurgery for teeth with an undeveloped root apex and periapical periodontitis caused by an abnormal central cusp fracture after failed nonsurgical treatment.

**Methodology:**

Eighty teeth in 78 patients were subjected to endodontic microsurgery. All patients were clinically and radiologically examined 1 year postoperatively. The data were statistically analyzed using SPSS 27.0 software.

**Results:**

Of the 80 teeth in 78 patients, periapical lesions had disappeared in 77 teeth at 1-year postoperative follow-up, with a success rate of approximately 96.3% (77/80). The efficacy of endodontic microsurgery was not affected by sex, age, extent of periapical lesions, and presence of the sinus tract. Between-group differences were not statistically significant (*P* > 0.05).

**Conclusions:**

Endodontic microsurgery can be an effective alternative treatment option for teeth with an undeveloped root apex and periapical periodontitis caused by an abnormal central cusp fracture after nonsurgical treatment failure.

## Background

Abnormal central cusps primarily occur in the mandibular second premolars, with a prevalence of approximately 2% in the Asian population and 1.29–3.6% in the Chinese population [1]. Several abnormal central cusps are worn or fractured by the time they are diagnosed. Bacteria commonly invade the root canal from the exposed pulp or dentinal tubule, thereby affecting root apex development by causing pulp infection and necrosis; this eventually results in a short root, a thin root canal wall, and unclosed apical foramen.

Nonsurgical treatment is generally the first choice of treatment for teeth with an unclosed root apex and periapical periodontitis [2]. Immature permanent teeth with pulp necrosis or periapical periodontitis are generally subjected to apexification, apical barrier technique or revascularization. These methods primarily aim to eliminate bacteria in the root canal to create a conducive environment to seal the root apex or promote root growth [2]. However, these procedures have certain failure rates, with 14% of the failures being caused by persistent infection [3]. When these treatment regimens fail, it seems pointless to reperform the treatment. As a result, a portion of the patients and dentists may lose faith during the treatment and opt for tooth extraction.

Endodontic microsurgery incorporates high magnification, ultrasonic root-end preparation and root-end filling. The development and widespread use of dental microscopes, microinstruments, biocompatible filling materials and cone-beam computerized tomography (CBCT) have facilitated the use of endodontic microsurgery as an alternate approach with predictable outcomes for endodontic diseases [4]. The root canals can be precisely obturated under a direct, clear vision to achieve high-quality obturation and improve the survival rate of those teeth.

The procedures mentioned above assist to achieve a better root canal obturation to isolate periapical tissue from bacteria in root canal or to promote tooth growth. Calcium silicate-based cements are the most used material in clinical settings owing to their excellent biocompatibility and sealing ability, which provide an extremely high success rate in endodontic microsurgery [5]. The effective obturation of the apical foramen can guarantee a successful endodontic microsurgery.

The present study aimed to determine if the endodontic microsurgery had an efficacy on teeth with an undeveloped root apex and periapical periodontitis that failed in nonsurgical treatment after one year follow-up, therefore, paving the clinical basis for the application of the endodontic microsurgery.

## Materials and methods

### Participants

Eighty teeth in 78 patients who had visited the Department of Endodontics of Suzhou Stomatological Hospital for the treatment of periapical periodontitis with an unclosed apical foramen from January 2021 to January 2022 were included in the study. These patients had previously undergone the apical barrier technique or revascularization, but low-density images still existed on the X-ray images of periapical tissue. The treatment outcomes were reassessed based on clinical symptoms and X-ray images after 6–18 months, including pain, presence of periapical lesions and sinus tract. The inclusion criteria were as follows: patients with (1) abnormal central cusp fractures that led to pulp necrosis, resulting in existence of low-density image on the X-ray image of periapical tissue, along with an undeveloped tooth apex; (2) bone destruction or the presence of clinical symptoms, although the apical barrier technique or revascularization was performed; and (3) no systemic diseases. The exclusion criteria were as follows: (1) presence of periodontitis and alveolar bone resorption beyond one-third of the apex or a crown-to-root ratio of less than 1:1; (2) complete absence of the bone plate on the labial (buccal) side of the tooth or penetration of periodontal lesions with periapical lesions; and (3) a root length of < 10 mm, with no value to preserve the tooth.

### Data collection

Information on the patients’ age, sex, and other parameters was collected. Radiographs were obtained preoperatively, immediately after surgery, and at 3 months, 6 months, and 1 year postoperatively. The patient’s clinical symptoms, radiological data and histopathological results were used to evaluate the transformation of the teeth and efficacy of endodontic microsurgery.

### Surgical method

**Fig. 1:**
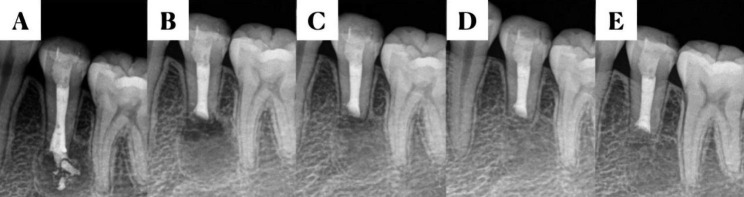
Changes in the periapical tissue after endodontic microsurgery upon the failure of the apical barrier technique. (**A**) Preoperative radiograph, (**B**) postoperative radiograph, and (**C**) follow-up radiographs after 3 months, (**D**) 6 months, and (**E**) 1 year

**Fig. 2:**
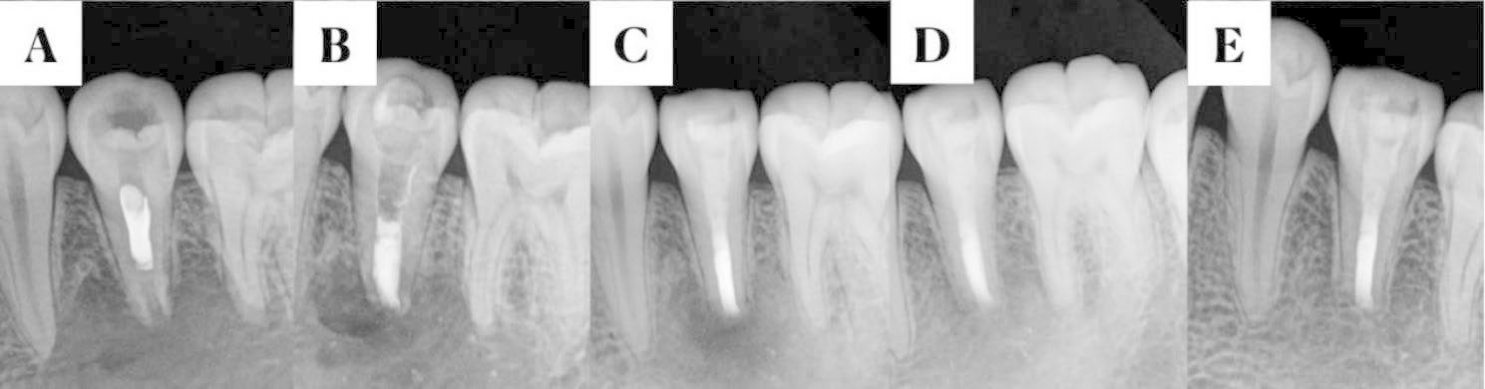
Changes in the periapical tissue radiographs after endodontic microsurgery upon revascularization failure. (**A**) Preoperative radiograph, (**B**) postoperative radiograph, and (**C**) follow-up radiographs after 3 months, (**D**) 6 months, and (**E**) 1 year

Preoperative radiography and CBCT were performed to comprehensively understand the morphology and length of the root, the location and extent of the periapical lesions, and their relationship with adjacent anatomical structures, including the mental foramen and mandibular nerve canal. All procedures were performed by the same endodontist. The procedure was performed in the following steps. After intra- and extraoral disinfection, the surgical area was locally anesthetized with 1:100,000 epinephrine in 4% articaine. A gingival sulcus incision and a proximal vertical incision were created under the lens of a dental microscope (Zumax 2380, Suzhou, China) on the labial (buccal) side of the affected tooth. The mucoperiosteal flap was subsequently turned upward. The apex of the affected tooth was identified, and a contra-angle handpiece (ImpactAir45, Kerr) with a high-speed fissure bur was used to create a bone opening. The lesion tissue was entirely scraped and sent for histopathological examination. Perpendicular to the central axis of the root, 1 mm of the root apex was removed using a long-handled fissure drill. The root was stained with methylene blue to determine whether there were microcracks under a high-magnification microscope. An ultrasonic instrument was used to perform a 3-mm root-end preparation with minimal damage to the dentin walls. Then, a 3-mm root-end filling was performed by using iRoot BP (Innovative Bioceramix, Canada). After confirming the absence of foreign bodies in the bone cavity, the bone walls were scratched to be filled with blood. The mucoperiosteal flap was restored and sewed by using a 6 − 0 absorbable suture. Finally, an X-ray image was immediately captured. The suture was removed 5–7 days postoperatively (Figs. 1 and 2).

### Evaluation criteria

The efficacy of the endodontic microsurgery was evaluated 1 year postoperatively based on the patients’ symptoms, clinical findings, and radiographs. (1) Complete healing was indicated by the complete or rough disappearance of the apical dark shadow, reappearance of the lamina dura, no significant abnormality in periodontal membrane width, and no clinical symptoms and signs. (2) Incomplete healing was considered when the apex showed scar tissue formation with no clinical pain, swelling, or sinus tract. (3) Uncertain healing was indicated by a decreased apical dark shadow that was still larger than normal. (4) Unsatisfactory healing was considered when imaging suggested that the size of the apical dark shadow remained the same or expanded. Radiographs were independently evaluated and classified by three endodontists based on radiological evaluation criteria. Before the evaluation, training and tests were performed by using standard samples, and a consistent diagnosis was ensured by using Cohen’s Kappa test analysis. After obtaining different evaluation results, the evaluators reached a consensus following a discussion.

### Statistical analyses

The SPSS 27.0 software package was used for data analysis. Nonparametric Wilcoxon’s signed rank test was used to analyze the effects of sex, age, distribution of teeth positions, presence of sinus tract, and extent of periapical lesion on the efficacy of endodontic microsurgery. Differences were considered statistically significant at *P* < 0.05.

## Results

The patients were aged 13–39 years (average age = 23 years). The study included 44 men and 34 women with 16 mandibular first premolars and 64 mandibular second premolars. Of these patients, 71 underwent the apical barrier technique and 9 underwent revascularization. As a result, 77 of the 80 affected teeth were successfully treated, with a success rate of 96.3% (77/80). The endodontic microsurgery outcomes were not statistically significant (*P* > 0.05) in terms of sex, age, distribution of teeth position, extent of periapical lesion, and presence of sinus tract (Table 1).

**Table 1:** Comparison of success rates between the groups

		Number of successful cases	Number of surgical failures	Total	Success rate
Age (years)	< 16	63	2	65	0.969
	> 16	14	1	15	0.933
Sex	Men	42	2	44	0.955
	Women	35	1	36	0.972
Sinus tract	Yes	54	2	56	0.964
	No	23	1	24	0.958
Teeth position	Mandibular first premolar	15	1	16	0.938
	Mandibular second premolar	62	2	64	0.969
Extent of periapical lesion	< 1 cm^2^	61	2	63	0.968
	> 1 cm^2^	16	1	17	0.941

## Discussion

Apexification is a classical method for treating teeth with an undeveloped root apex and periapical periodontitis by stimulating calcification and producing the apical closure. It may involve one or more appointments per month to place calcium hydroxide inside the root canal to eliminate the intracanal infection. Although the success rate of apexification has been reported to be desirable [6], it has several limits in clinical settings, including long treatment course, unpredictability between apical closure and treatment time, and poor patient follow-up compliance because of the long treatment course. Calcium hydroxide is the first choice for apexification. However, long periods of calcium hydroxide in contact with the root canal walls will dissolve collagen and other bioactive matrix components of mineralized dentin. The resistance to root fracture will reduce drastically [7] and may even prevent it from further development [8]. Therefore, the overall prognosis may be affected by apexification because of these factors.

Pulp revascularization can be defined as the vascularization of an immature permanent tooth with an infected necrotic pulp and periapical periodontitis/ abscess [9]. It can promote the root development and reinforcement of dentinal walls by deposition of hard tissue. Revascularization significantly increases root length and thickness compared with apexification, and thus, this advantage reduces the possibility of postoperative root fracture. Nevertheless, other challenges include different root shapes, time of failure, or intracanal changes [10]. The choice of root canal disinfectant may directly or indirectly affect the survival of stem cells in the periapical tissue, making it difficult to accurately predict its efficacy [11, 12]. The persistence of bacteria usually confuses endodontists. A meta-analysis and systematic review by Tong et al. [13] revealed that current evidence is insufficient to provide definitive conclusions on revascularization predictability. The age of the patient should also be considered before treatment. Immature permanent teeth between the ages of 9 and 18 years are more suitable for revascularization, and the prognosis may be worse with age increasing [14].

The apical barrier technique involves the addition of a biocompatible artificial barrier directly in the apical region after controlling infection to achieve immediate root canal obturation. It significantly reduces the treatment time and has become one of the preferred techniques for treating teeth with an unclosed apex foramen.

Whether apexification, apical barrier technique, or revascularization is performed for immature permanent teeth with periapical periodontitis, a certain rate of failure exists because of the difficulty of eliminating bacteria in the root canal, the lesion of periapical periodontitis, and patients’ age and general condition. Once apexification fail, apical barrier technique and revascularization can be used as an alternative. While when they fail again, it is difficult to gain access from the crown because calcium silicate-based cements would solidify. Undergoing the same procedure again would mean losing more dentine along with an unpredictable result. In such cases, endodontic microsurgery could be another choice to preserve teeth. The advantage of endodontic microsurgery is that it promotes tissue healing by tightly sealing the root canal system under the direct vision and creating an effective barrier preventing microorganisms from entering the periapical tissue. A recent retrospective analysis [15] on endodontic microsurgery revealed that the affected teeth have a long-term survival rate of 95.2%.

Calcium silicate-based cement plays an indispensable role in such treatments, contributing to favorable biocompatibility, osteoconductivity, sealing ability, and clinical operability [5, 16]. It also contains certain amounts of oxide compounds known to potentially exhibit antimicrobial activities [17]. Materials such as iRoot BP have been found to enhance MC3T3-E1 preosteoblast activity in the acidic environment formed by anaerobic bacteria, facilitating periapical healing [7, 12, 18].

All 80 affected teeth included in this study were permanent premolars with abnormal central cusp fractures and unclosed apical foramen, resulting in periapical periodontitis. All teeth had been subjected to the apical barrier technique or revascularization before. In cases which the root canal infection is not effectively controlled during the previous treatment follow-up, endodontic microsurgery may be considered the last alternative to preserve tooth. The classical 3-mm apicoectomy generally required in endodontic microsurgery was not performed because the occurrence of the lateral canal and canal divergence were less likely. Only 1-mm apicoectomy was performed to ensure an adequate crown-to-root ratio. Subsequently, 3-mm root-end preparation and 3-mm root-end filling were performed.

In two of the failed cases, the existence of root fracture was confirmed after tooth extraction. Reduction of dentin thickness is one of the most common reasons [19–21]. The resistance of immature premolars, already unsatisfactory due to the short root canals and the thin dentinal walls, may be further reduced after apicoectomy and root-end preparation. Maintaining balance between bacterial removal and preservation of dental tissue require further research. After expelling leaky canal, missing canal, anatomical complexity, over filling and root fracture, the last failed case was diagnosed as unexplained failure.

## Conclusion

At 1-year postoperative follow-up, only 3 of the 80 affected teeth failed, with a success rate of approximately 96.3%. Nonsurgical treatment is absolutely the first choice for teeth with an undeveloped root apex and periapical periodontitis. After failure of these nonsurgical treatments, however, the access of root canal may have been blocked by calcium silicate-based cements. In these cases, endodontic microsurgery for preserving such teeth is highly feasible. However, the roots of the affected teeth were undeveloped. Long-term observation is required to understand whether the short length of the root or the thin dentinal wall decreases root fracture resistance after endodontic microsurgery.

## Data Availability

The datasets generated and/ or analyzed during the current study are not publicly available due to privacy or ethical restrictions but are available from the corresponding author on reasonable request.

## Abbreviations

CBCT: Cone-beam computerized tomography

## References

[1] Rao Y, Guo L, Hu T (2010). Multiple dens Evaginatus of Premolars and Molars in chinese dentition: a Case Report and Literature Review. Int J Oral Sci.

[2] Murray PE (2023). Review of guidance for the selection of regenerative endodontics, apexogenesis, apexification, pulpotomy, and other endodontic treatments for immature permanent teeth. Int Endod J.

[3] Wikström A, Brundin M, Romani Vestman N, Rakhimova O, Tsilingaridis G (2022). Endodontic pulp revitalization in traumatized necrotic immature permanent incisors: early failures and long-term outcomes—A longitudinal cohort study. Int Endod J.

[4] Kim S, Kratchman S (2006). Modern endodontic surgery concepts and practice: a review. J Endod.

[5] Chan S, Glickman GN, Woodmansey KF, He J (2020). Retrospective analysis of Root-end microsurgery outcomes in a Postgraduate Program in Endodontics using calcium silicate–based Cements as Root-end filling materials. J Endod.

[6] Al Ansary MAD, Day PF, Duggal MS, Brunton PA (2009). Interventions for treating traumatized necrotic immature permanent anterior teeth: inducing a calcific barrier & root strengthening. Dent Traumatol.

[7] Arikan V. Comparative evaluation of four endodontic biomaterials and calcium hydroxide regarding their effect on fracture resistance of simulated immature teeth. Eur J Paediatr Dent. 2020;:23–8. 10.23804/ejpd.2020.21.01.05 32183524

[8] Hargreaves KM, Diogenes A, Teixeira FB (2013). Treatment Options: biological basis of regenerative endodontic procedures. J Endod.

[9] Xie Z, Shen Z, Zhan P, Yang J, Huang Q, Huang S (2021). Functional Dental Pulp Regeneration: Basic Research and clinical translation. Int J Mol Sci.

[10] Lee C, Song M (2022). Failure of regenerative endodontic procedures: case analysis and subsequent treatment options. J Endod.

[11] Trevino EG, Patwardhan AN, Henry MA, Perry G, Dybdal-Hargreaves N, Hargreaves KM (2011). Effect of Irrigants on the survival of human stem cells of the apical papilla in a platelet-rich plasma Scaffold in Human Root Tips. J Endod.

[12] Yadav P, Pruthi PJ, Naval RR, Talwar S, Verma M (2015). Novel use of platelet-rich fibrin matrix and MTA as an apical barrier in the management of a failed revascularization case. Dent Traumatol.

[13] Tong HJ, Rajan S, Bhujel N, Kang J, Duggal M, Nazzal H (2017). Regenerative endodontic therapy in the management of Nonvital Immature Permanent Teeth: a systematic review—outcome evaluation and Meta-analysis. J Endod.

[14] Estefan BS, El Batouty KM, Nagy MM, Diogenes A (2016). Influence of age and apical diameter on the success of endodontic regeneration procedures. J Endod.

[15] Huang S, Chen N-N, Yu VSH, Lim HA, Lui J-N (2020). Long-term success and survival of Endodontic Microsurgery. J Endod.

[16] Kim Y, Lee D, Kye M, Ha Y-J, Kim S-Y (2022). Biocompatible Properties and Mineralization potential of Premixed Calcium Silicate-Based cements and fast-set calcium silicate-based cements on human bone marrow-derived mesenchymal stem cells. Mater (Basel).

[17] Janini ACP, Bombarda GF, Pelepenko LE, Marciano MA (2021). Antimicrobial activity of Calcium Silicate-Based Dental materials: a Literature Review. Antibiot (Basel).

[18] Tian J, Zhang Y, Lai Z, Li M, Huang Y, Jiang H (2017). Ion Release, Microstructural, and Biological Properties of iRoot BP plus and ProRoot MTA exposed to an acidic environment. J Endod.

[19] Makeeva IM, Byakova SF, Novozhilova NE (2016). Risk factors for vertical root fractures after endodontic treatment. Stomatologiia (Mosk).

[20] Patel S, Bhuva B, Bose R (2022). Present status and future directions: vertical root fractures in root filled teeth. Int Endod J.

[21] Yan W, Jiang H, Deng Z, Paranjpe A, Zhang H, Arola D (2021). Shrinkage strains in the dentin of endodontically treated Teeth with Water loss. J Endod.

